# The metabolites mainly composed of lipids in tongue coating are non-invasive potential biomarkers for chronic gastritis

**DOI:** 10.1038/s41598-024-68261-6

**Published:** 2024-07-30

**Authors:** Zhiyuan You, Junhong Lu, Yifeng Xu, Renling Zhang, Zhujing Zhu, Yiqin Wang, Yiming Hao

**Affiliations:** 1 Shanghai Key Laboratory of Health Identification and Assessment/Laboratory of TCM Four Diagnostic Information, Shanghai University of Traditional Chinese Medicine, 1200 Cailun Road, Pudong New Area, Shanghai, 201203 China; 2 Longhua Hospital Shanghai University of Traditional Chinese Medicine, 725 South Wanping Road, Shanghai, China

**Keywords:** Metabolomics, Gastroscopy indicators, Gastric mucosal pathology, Non-invasive diagnosis, Gastrointestinal diseases, Oesophagogastroscopy

## Abstract

The changes in tongue coating metabolites in patients with chronic gastritis (CG) under different gastroscopy indicators were analyzed, and these metabolites were screened for potential non-invasive biomarkers to assist in the diagnosis of chronic gastritis. The technology of gas chromatography and liquid chromatography combined with mass spectrometry has been used to more comprehensively detect tongue coating metabolites of 350 CG patients. Spearman correlation analysis and random forest algorithm were used to screen metabolites that can serve as potential biomarkers. Compared with healthy individuals, CG group showed significant changes in the content of 101 metabolites, with an increase in the content of 54 metabolites and a decrease in the content of 47 metabolites. These differential metabolites are mainly composed of 47 lipids and lipid like substances. 1 metabolite was associated with bile reflux, 1 metabolite was associated with gastric mucosal erosion, 10 metabolites were associated with atrophy, 10 metabolites were associated with intestinal metaplasia, and 3 metabolites were associated with *Helicobacter pylori* infection. The ROC model composed of 5 metabolites can distinguish between CG group and healthy individuals, with an accuracy of 95.4%. The ROC model composed of 5,6-Dihydroxyindole can distinguish between chronic superficial gastritis group and chronic atrophic gastritis group, with an accuracy of 75.3%. The lipids and lipid like metabolites were the main abnormal metabolites in patients with chronic gastritis. It was worth noting that the content of Sphinganine 1-phase, 4-Ipomenol, and Nervonic acid in tongue coating increased, and the content of 1-Methyladenosine and 3-Hydroxycapric acid decreased, which helped to identify CG patients. The decrease in the content of 5,6-dihydroxyindole reminded patients that the development trend of CG was shifting from superficial to atrophic or even intestinal metaplasia. The detection of these metabolic markers of tongue coating was expected to be developed as a non-invasive and convenient technology in the future to assist us in monitoring and diagnosing the occurrence and development of CG.

## Introduction

Nowadays, with the faster pace of people’s lives and the change of people’s dietary structure, more and more people are suffering from digestive diseases. Chronic gastritis (CG) as one of the most common digestive diseases, has affected more than half the world's population in recent years. The development of CG generally progresses from superficial gastritis to atrophic gastritis, and atrophic gastritis has a certain chance of transforming into gastric cancer^1^. Therefore, early detection and treatment of CG are of great significance in preventing further injury and improving the quality of life. Although gastroscopy is currently the most reliable method for diagnosing CG, it cannot be performed frequently in a short time^2^. Thus, finding a noninvasive and repeatable monitoring method to observe the progress of CG is one of the main directions of our research.

According to the theory of traditional Chinese medicine (TCM), the formation of tongue coating is influenced by the physiological function of the stomach. Therefore, TCM doctor often diagnoses gastric disease by observing changes in the tongue^3^, which is non-invasive and repeatable. Some researchers have used liquid chromatography-mass spectroscopy technology to discover differences in tongue coating metabolites between CG patients and healthy individuals^4,5^. However, these studies only used one metabolomics detection method and had a small sample size, which resulted in incomplete detection of metabolites. Meanwhile, studies on the correlation of tongue coating metabolites and gastroscopic indexes in CG patients have not been reported.

The gas chromatography-time-of-light-mass spectroscopy (GC-TOF-MS) and ultra high-performance liquid chromatography-Q-exactive-mass spectroscopy (UHPLC-QE-MS) are the two most commonly used techniques for metabolomics detection. The two detection methods target different levels of metabolite molecular weight, volatility, and solubility^6^. Therefore, for the complex composition of metabolites in tongue coating, a combination of two methods can be used to achieve a more comprehensive detection.

Therefore, this study used a combination of GC-TOF-MS and UHPC-QE-MS to detect tongue coating samples from 350 CG patients, and then used bioinformatics and statistical methods to identify potential metabolic markers in the development of CG.

## Materials and methods

### Samples

This study diagnosed 350 patients with CG through gastroscopy and gastric mucosal pathological examination at Longhua Hospital. Additionally, 50 healthy controls from Shanghai University of TCM were selected. The healthy controls had no history of stomach disease or current stomach discomfort. The health of the control group was confirmed by a comprehensive health assessment including haematological cytology, liver and renal function, blood glucose, blood lipids, tumour markers, blood pressure, colour Doppler ultrasound of the neck and abdomen, and 13C urea breath test. The participants included in this study were Han Chinese (self-reported), and the sample was collected from December 2018 to October 2019. Gastroscopy and gastric mucosal pathology were performed promptly after obtaining tongue coating specimens from the participants.

### Ethics approval and statement

The ethics approval for the study was granted by the Ethics Committee of Shanghai University of TCM, with the approval obtained in December 2018. Prior to sample collection, all the participants provided their informed consent by signing consent agreements, indicating their voluntary participation in the study. The experimental procedures were carried out in strict accordance with the Declaration of Helsinki and local laws.

### Criteria

With reference to the first and second editions of the Kyoto Classification of Gastritis, patients were subjected to endoscopic examination and biopsies were performed from suspected lesions of gastric antrum, gastric angle, gastric body, and cardia^7^. The gastroscopic report includes six gastroscopic and pathological indicators: bile reflux, gastric mucosal erosion, inflammatory activity, atrophy (mild, moderate, or severe), intestinal metaplasia (mild, moderate, or severe), and *Helicobacter pylori* (Hp) infection (mild, moderate, or severe).

The inclusion and exclusion criteria followed our previous study which were shown in Supplementary file and Fig. S1^8^.

### Tongue coating collection

The tongue coating was collected using our previous collection method^8^. The specific acquisition method was shown in Supplementary file.

### Metabolomics testing

In the GC-TOF-MS testing, the reagents used were listed in Table S3a. The experimental steps were presented in Supplementary file. The original data were analyzed by using Chroma TOF (V 4.3x, LECO) software^9^. The mass spectrum and retention index identification of metabolites were performed by using the LECO-Fiehn Rtx5 database. The peaks detected in QC samples were less than half of the peaks were removed in QC samples with RSD > 30%^10^.

In the UHPLC-QE-MS testing, the reagents used were listed in Supplementary Table S3b. The experimental steps were presented in Supplementary file. The cutoff value for annotation was set to 0.3^11^.

### Statistical analysis

The Simca-p+13.0 software (Umea, Sweden) was used for OPLS-DA. To further validate the model, a drive substitution experiment was carried out. The significance of metabolite differences between two groups was determined using various statistical criteria, including the false discovery rate (FDR) of the rank-sum test, P-values (*P* < 0.05), variable importance in the projection (VIP) of the first principal component of the OPLS-DA model (VIP > 1)^11^, similarity value (SV) identified through GC-TOF-MS (SV > 700)^12^, and MS2 score obtained from UHPLC-QE-MS detection (MS2 score > 0.6)^13^. Metabolite peak areas' mean values were compared between the two groups, and the log fold change (FC) value was subsequently calculated to assess the magnitude of differences.

The significantly different metabolites of the two groups were randomly combined, and the receiver operating characteristic (ROC) curve was used to evaluate model accuracy. Finally, the combination with the highest AUC value was selected as the diagnostic model.

The Spearman algorithm was used to analyze correlation between gastroscopic indicators and differential metabolites. The Corr matrix, correlation P-values were calculated for subsequent analysis and graphically plotted.

Among the metabolites that have significant correlation with gastroscopic indicators, Analysis of variance (ANOVA) was used to compare the standardized peak areas of these metabolites in patients with chronic gastritis when there were different levels of pathological indicators.

We performed the total ion flow normalisation method on the measured peaks and tested for normality. The data did not conform to a normal distribution, so metabolites with significant differences were screened by the rank sum test (see “Materials and methods”). Using significantly different metabolites that can be retrieved in the Fiehn database, ROC curves were plotted for the CG group versus the healthy control group and AUC values were obtained. The AUC values were sorted in descending order, and the top 10 metabolites with the highest AUC values in the ROC curves were selected for random combination. The ROC curves of the CG group and the healthy control group were plotted again. The ROC curve with the highest AUC value and involving the lowest number of differential metabolites was derived. As a diagnostic model for the CG group versus healthy controls.

### Ethics approval and consent to participate

The Ethics Committee of Shanghai University of TCM approved our study. The study obtained informed consent from all participants.

## Results

### Basic information analysis results

It can be seen from Table S1 that there was no significant difference in the sex ratio between the CG group and healthy controls, but there was significant difference in the age. Therefore, the orthogonal projections to late structures-discriminant analysis (OPLS-DA) were used to observe the interference of age factors between the two groups. From Fig. S2, it can be shown that the metabolites were not significantly distinguished, which suggested that age does not affect the reliability of our results.

**Figure 1:**
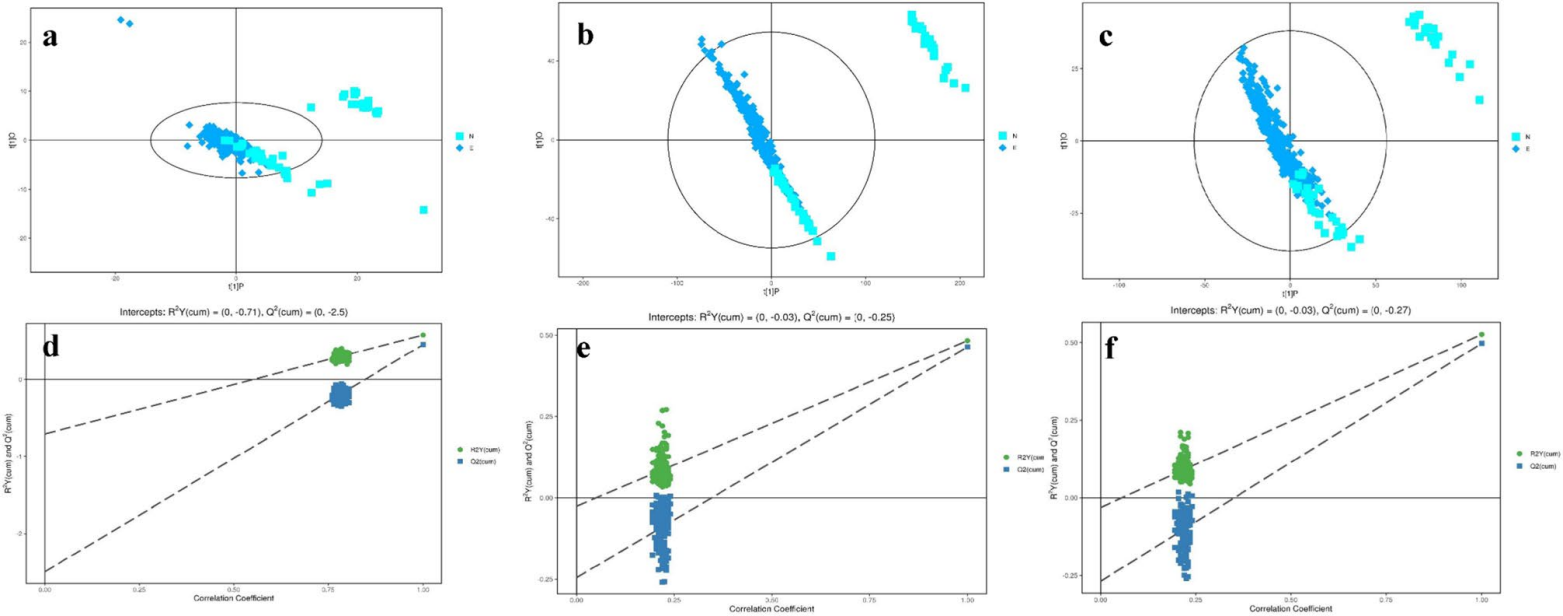
OPLS-DA analysis of the chronic gastritis group and healthy controls. (**a**) GC-TOF-MS scatterplot of OPLS-DA model scores. (**b**) UHPLC-QE-MS positive ion modes scatterplot of OPLS-DA model scores. (**c**) UHPLC-QE-MS negative ion modes scatterplot of OPLS-DA model scores. (**d**) GC-TOF-MS plot of permutation test results for the OPLS-DA model. (**e**) UHPLC-QE-MS positive ion modes plot of permutation test results for the OPLS-DA model. (**f**) UHPLC-QE-MS negative ion modes plot of permutation test results for the OPLS-DA model.

The horizontal axist[1]P represents the predicted component scores of the first principal component, while the vertical axis t[1]O represents the scores of the orthogonal component. The scatter plot uses different shapes and colors to represent distinct groups. According to the results from the OPLS-DA score plot, it is evident that the differentiation between sample groups is significant, and the samples generally fall within the 95% confidence interval (show in Fig. 1).

The Q2 values of the random model were consistently lower than those of the original model. The intercept of the regression line between Q2 and the vertical axis was less than zero. Simultaneously, as the permutation retention rate gradually decreased, the proportion of permuted Y variables increased, leading to a gradual decline in the Q2 of the random model. This indicates that the original model exhibits good robustness and does not show signs of overfitting (show in Fig. 1).

### Different metabolic MS peaks in tongue coating samples

**Figure 2:**
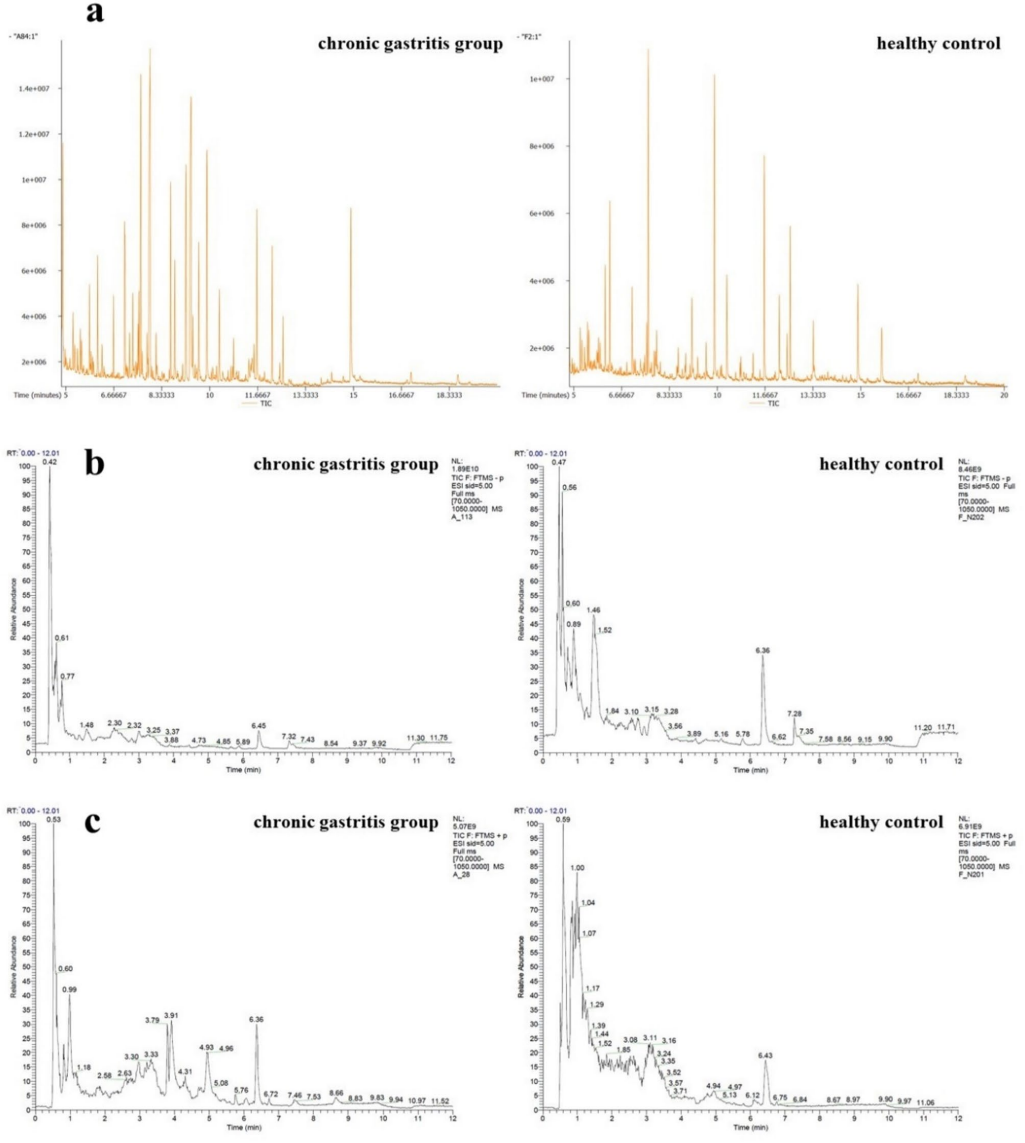
Mass spectrum peaks of chronic gastritis group and healthy control group. (**a**) GC-TOF-MS, (**b**) UHPLC-QE-MS negative ion modes, (**c**) UHPLC-QE-MS positive ion modes. As shown in (**a**–**c**), there were some different mass spectrum peaks between the chronic gastritis patients and healthy control people.

As depicted in Fig. 2a–c, discernible differences in MS peaks were observed between CG patients and healthy controls.

Applying the criteria of P-value < 0.05 and VIP > 1, the analysis using GC-TOF-MS identified 32 peaks in the tongue coating of CG patients (20 increased and 12 decreased). Similarly, UHPLC-QE-MS positive analysis revealed 98 peaks (62 increased and 36 decreased), while UHPLC-QE-MS negative analysis detected 14 peaks (10 increased and 4 decreased).

When employing the criteria of similarity (SV > 700) or MS2 score (> 0.6), GC-TOF-MS analysis unveiled 19 peaks (11 increased and 8 decreased) in the tongue coating of CG patients. For UHPLC-QE-MS positive analysis, 72 peaks were identified (36 increased and 36 decreased), and UHPLC-QE-MS negative analysis revealed 10 peaks (7 increased and 3 decreased). These findings highlight the distinct metabolic profiles present in the tongue coating of CG patients compared to healthy controls.

### Different metabolites analysis

In Table 1, 85 metabolites were divided into 11 categories, of which the largest category was benzenoids (2 metabolites), lipids and lipid-like molecules (47 metabolites), organic acids and derivatives (13 metabolites), organic nitrogen compounds (6 metabolites), organic oxygen compounds (4 metabolites), and organoheterocyclic compounds (8 metabolites). There was one metabolite in homogeneous non-metal compounds, hydrocarbons, nucleosides, nucleotides, and analogues, organosulfur compounds, and phenylpropanoids and polyketides.

**Table 1:** Identification of significant different metabolites in tongue coating by comparison of chronic gastritis patients and healthy controls.

Metabolite name	RT (min)	M/Z	Chronic gastritis Mean	Healthy control Mean	*P*	VIP	Log fold change	Detection method
*Lipids and lipid-like molecules*								
Arachidonic acid	37.582	303	17.019	4.430	1.509E−09	1.569	1.942	LC−
Prostaglandin D2	46.555	351	1.937	1.098	8.949E−10	1.657	0.819	LC −
9,10-DHOME	81.276	313	0.133	0.634	0.001	1.256	− 2.256	LC −
Azelaic acid	359.713	187	0.191	0.796	1.889E−12	1.731	− 2.062	LC −
Ethyl oleate	41.892	309	0.692	0.332	4.658E−12	1.241	1.060	LC −
8,11,14-Eicosatrienoic acid	37.965	305	0.760	0.229	1.003E−12	1.364	1.727	LC −
Nervonic acid	37.630	365	0.309	0.128	6.539E−15	1.521	1.275	LC −
Tetracosanoic acid	37.748	367	0.561	0.357	4.645E−07	1.040	0.651	LC −
Foeniculoside VII	71.454	349	0.517	1.452	2.235E−04	1.168	−1.490	LC +
Glycerol tripropanoate	61.345	261	1.163	2.718	0.002	1.078	− 1.225	LC +
Solavetivone	115.309	219	0.950	0.134	7.717E−10	1.408	2.826	LC +
Patchoulenone	61.062	219	11.219	3.010	9.843E−06	1.438	1.898	LC +
Gibberellin A79	71.856	365	0.064	0.152	2.553E−04	1.063	− 1.247	LC +
Oleic acid	33.290	283	0.263	0.421	9.784E−06	1.132	− 0.678	LC +
Dexamethasone	77.202	393	0.262	1.323	1.264E−04	1.249	− 2.335	LC +
3-*O*-Methylniveusin A	77.445	409	0.044	0.277	2.619E−07	1.373	− 2.641	LC +
Hoduloside VII	248.651	932	0.926	0.579	2.553E−05	1.398	0.678	LC +
Fucoxanthin	259.139	659	0.077	0.392	2.217E−10	1.576	− 2.344	LC +
* N*-Cyclopropyl-trans-2-cis-6-nonadienamide	46.068	194	0.174	0.114	2.907E−07	1.364	0.611	LC +
3-Hydroxyisovalerylcarnitine	57.688	262	0.119	1.688	4.202E−09	1.457	− 3.830	LC +
Lyciumoside III	142.803	649	0.025	2.219	3.956E−11	1.524	− 6.491	LC +
Eicosadienoic acid	33.311	309	0.795	3.662	4.891E−10	1.584	− 2.203	LC +
3beta-Acetoxy-11alpha-methoxy-12-ursen-28-oic acid	193.697	543	0.092	0.044	5.348E−12	1.400	1.071	LC +
Antibiotic X 14889C	257.759	615	0.082	0.406	7.246E−11	1.561	− 2.308	LC +
2-Ethyl-2-Hydroxybutyric acid	58.656	133	0.727	2.302	6.578E−06	1.406	− 1.663	LC +
Palmitoyl glucuronide	293.193	419	0.408	0.261	8.303E−06	1.422	0.648	LC +
Physalin O	314.512	529	0.129	0.082	3.070E−05	1.426	0.663	LC +
Desglucocoroloside	220.265	505	0.030	0.017	4.319E−13	1.078	0.792	LC +
3-Methylglutarylcarnitine	84.851	290	0.257	0.182	0.008	1.289	0.502	LC +
Smilanippin A	113.780	725	0.594	0.375	2.235E−04	1.408	0.662	LC +
PS(16:1(9Z)/18:4(6Z,9Z,12Z,15Z))	207.222	754	0.069	0.573	2.189E−13	1.592	− 3.047	LC +
CPA(18:0/0:0)	278.918	421	0.210	0.131	1.435E−04	1.368	0.682	LC +
Cucurbitacin I 2-glucoside	205.358	677	1.712	1.092	1.325E−04	1.092	0.648	LC +
Glycerol tributanoate	125.367	303	0.170	0.563	1.158E−05	1.081	− 1.728	LC +
Fasciculic acid C	116.186	710	0.470	1.367	3.248E−07	1.406	− 1.539	LC +
Hebevinoside I	38.382	809	0.097	0.365	2.113E−06	1.375	− 1.917	LC +
2,4,6-Octatriynoic acid	440.442	133	0.296	0.478	6.027E−12	1.127	− 0.692	LC +
Lycoperoside D	154.407	740	0.568	11.651	8.613E−06	1.352	− 4.358	LC +
Chondrillasterol 3-[glucosyl-(1->4)-glucoside]	97.620	737	0.687	1.821	2.655E−11	1.488	− 1.406	LC +
Physapruin B	227.103	603	0.099	0.058	5.799E−08	1.112	0.778	LC +
Traumatic acid	281.345	229	0.497	0.421	0.047	1.196	0.239	LC +
Lactosylceramide (d18:1/26:0)	31.388	1003	0.003	1.964	2.231E−16	1.364	− 9.313	LC +
Sphinganine 1-phosphate	51.580	382	0.166	0.069	2.606E−14	1.554	1.256	LC +
3-Benzoyloxy-11-oxo-12-ursen-28-oic acid	34.377	575	2.140	1.439	0.002	1.278	0.573	LC +
Momordicoside K	69.766	649	0.674	1.394	2.049E−11	1.508	− 1.049	LC +
PI(22:5(4Z,7Z,10Z,13Z,16Z)/16:0)	244.507	886	0.154	0.528	7.818E−08	1.368	− 1.779	LC +
Vinaginsenoside R3	84.853	932	0.466	1.065	1.241E−09	1.445	− 1.192	LC +
*Organic acids and derivatives*								
Glycolic acid	5.611	147	0.001	0.002	0.005	1.229	− 0.518	GC
Pantothenic acid	11.358	103	4.356E-04	2.233E-04	1.183E−08	1.143	0.964	GC
3-Hydroxycapric acid	83.306	187	0.214	0.441	3.691E−15	1.383	− 1.043	LC −
Bortezomib	285.056	407	0.080	0.811	4.942E−05	1.026	− 3.350	LC +
Ustiloxin D	287.091	495	0.044	0.450	5.519E−04	1.239	− 3.339	LC +
Symmetric dimethylarginine	158.848	203	4.835	6.288	1.124E−12	1.032	− 0.379	LC +
Oseltamivir	314.576	313	0.145	0.096	2.488E−04	1.397	0.593	LC +
Arginyl-Arginine	296.631	331	2.106	1.462	0.045	1.439	0.526	LC +
Arginyl-Histidine	166.723	312	0.423	0.284	0.010	1.342	0.574	LC +
Dihydro-3-(2-octenyl)-2,5-furandione	281.391	211	0.127	0.101	0.012	1.196	0.323	LC +
*N*-Acetylleucine	265.067	174	0.154	0.099	2.133E−04	1.231	0.632	LC +
Valyl-Isoleucine	221.874	231	0.092	0.032	3.496E−12	1.161	1.498	LC +
l-cis-3-Amino-2-pyrrolidinecarboxylic acid	75.782	131	0.032	0.683	1.979E−12	1.399	− 4.437	LC +
*Organoheterocyclic compounds*								
1-(1-Pyrrolidinyl)-2-propanone	53.003	128	1.823	2.480	7.682E−13	1.031	− 0.444	LC +
Pyrimidine	112.505	81	0.097	0.320	1.230E−04	1.275	− 1.728	LC +
5,6-Dihydroxyindole	45.066	150	0.251	0.169	1.267E−05	1.128	0.574	LC +
N1-Methyl-4-pyridone-3-carboxamide	79.822	153	0.212	0.176	0.032	1.002	0.269	LC +
2-Methylpiperidine	299.990	100	3.360	2.030	1.331E−06	1.371	0.727	LC +
Alpha-Carboxy-delta-decalactone	125.959	215	0.080	0.336	9.623E−07	1.087	− 2.065	LC +
I-Urobilin	174.113	591	0.121	0.476	3.184E−06	1.214	− 1.974	LC +
Garcimangosone C	282.076	413	0.857	0.656	0.048	1.287	0.387	LC +
*Organic nitrogen compounds*								
1-Butylamine	268.847	74	12.807	7.239	2.310E−08	1.402	0.823	LC +
Nervonyl carnitine	237.324	102	636.919	429.936	1.702	1.351	0.567	LC +
Isobutylpropylamine	231.650	116	0.444	0.294	7.682E−04	1.294	0.596	LC +
2-Diethylaminoethanol	300.009	118	196.915	117.700	2.547E−06	1.441	0.742	LC +
Dipyridamole	47.761	253	0.366	9.287	0.016	1.060	− 4.666	LC +
l-Histidinol	62.697	142	0.424	0.160	2.656E−07	1.289	1.406	LC +
*Organic oxygen compounds*								
Glycerol	7.006	85	1.092E-04	2.011E-04	2.605E−08	1.884	− 0.882	GC
4-Ipomeanol	72.941	167	0.405	0.072	1.036E−17	1.626	2.499	LC −
Leonuriside A	372.037	333	0.012	0.235	6.978E−12	1.365	− 4.293	LC +
Trimethylaminoacetone	206.235	116	0.123	0.095	2.258E−04	1.324	0.380	LC +
*Benzenoids*								
Dictagymnin	60.998	203	0.237	0.059	8.304E−05	1.007	2.008	LC +
Salmeterol	57.091	416	0.458	1.005	4.693E−09	1.466	− 1.134	LC +
*Homogeneous non-metal compounds*								
hydroxylamine	5.968	85	6.208E-04	0.001	2.224E−08	1.240	− 0.834	GC
*Nucleosides, nucleotides, and analogues*								
1-Methyladenosine	185.868	282	0.375	0.692	3.673E−14	1.015	− 0.884	LC +
*Phenylpropanoids and polyketides*								
Sofalcone	285.882	451	0.064	0.639	9.614E−05	1.259	− 3.311	LC +
*Organosulfur compounds*								
(Â±)-2-Pentanethiol	32.769	105	0.419	0.279	1.489E−05	1.195	0.590	LC +
Hydrocarbons								
1-Methyl-1,3-cyclohexadiene	32.571	95	0.476	0.315	3.084E−09	1.290	0.595	LC +
*Others*								
6-Deoxyglucitol	10.173	117	1.979E−04	1.416E−04	1.789E−04	2.679	0.483	GC
Myristic acid	10.642	117	9.197E−04	0.002	1.892E−09	1.264	− 1.213	GC
Octadecanol	12.158	327	2.588E−05	3.633E−05	6.106E−05	1.036	− 0.489	GC
1,2,4-Benzenetriol	9.344	124	0.006	0.003	0.002	2.238	0.835	GC
Threitol	8.562	191	4.846E−05	1.651E−04	4.677E−11	1.286	− 1.769	GC
2,4-Dichloro-1-(2-chloroethenyl)-benzene	7.925	170	0.012	0.008	0.011	2.553	0.645	GC
Asparagine dehydrated	8.628	110	2.811E−04	2.228E−04	0.002	1.493	0.335	GC
Conduritol-beta-expoxide	11.541	103	0.005	0.003	1.260E−05	1.674	0.696	GC
Methylmaleic acid	7.923	170	0.012	0.008	0.037	2.782	0.601	GC
4-Methylcatechol	7.928	170	0.012	0.008	0.036	2.120	0.607	GC
Glucose-6-phosphate major	12.806	111	5.894E−05	4.413E−05	1.142E−04	2.808	0.417	GC
Uridine minor	13.354	145	0.002	0.001	0.015	2.825	0.387	GC
Methylmalonic acid	6.698	85	7.146E−05	1.287E−04	9.368E−08	1.764	− 0.849	GC
5-Hydroxynorvaline	8.88	85	9.138E−04	0.002	2.404E−06	1.157	− 0.977	GC
Sulfuric acid	6.326	117	1.023E−04	7.394E−05	0.003	1.921	0.469	GC
(10S,11R)-Pterosin C 4-glucoside	282.232	397	15.388	11.382	0.024	1.345	0.435	LC +

GC, GC-TOF-MS; LC +, UHPLC-QE-MS positive ion; LC −, UHPLC-QE-MS negative ion.

### Diagnostic model of CG

**Figure 3:**
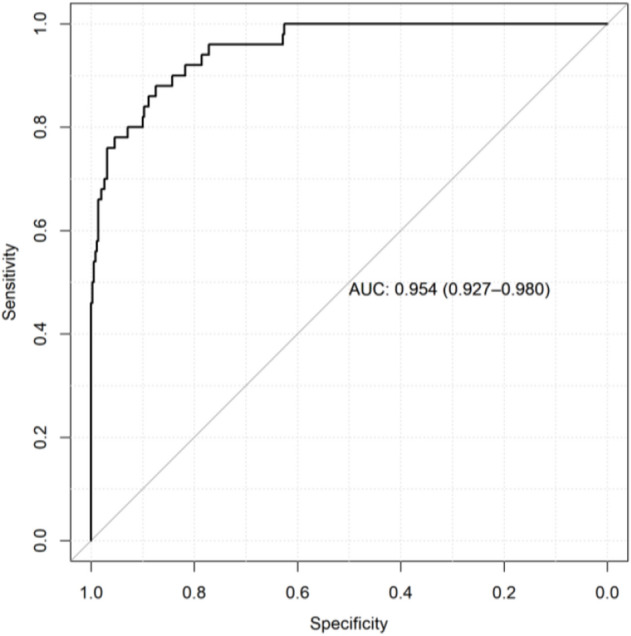
Diagnostic model of tongue coating metabolites in patients with chronic gastritis. The accuracy of the diagnostic model is 95.4%, the specificity is 87.4%, and the sensitivity is 88.0%.

As shown in Fig. 3, the diagnostic model was constructed by identifying distinctive metabolites of 350 CG patients and 50 healthy individuals. The optimal diagnostic model comprised 5 tongue coating metabolites. Comparative analysis with healthy controls revealed significant upregulation of 4-Ipomeanol, Nervonic acid, and Sphinganine 1-phosphate in CG patients, whereas 1-Methyladenosine and 3-Hydroxycapric acid exhibited significant downregulation in CG group. These findings underscore the potential of these identified metabolites as valuable markers for distinguishing CG patients from healthy individuals in the diagnostic context.

### Correlation between different metabolites and gastroscopy indexes

**Figure 4:**
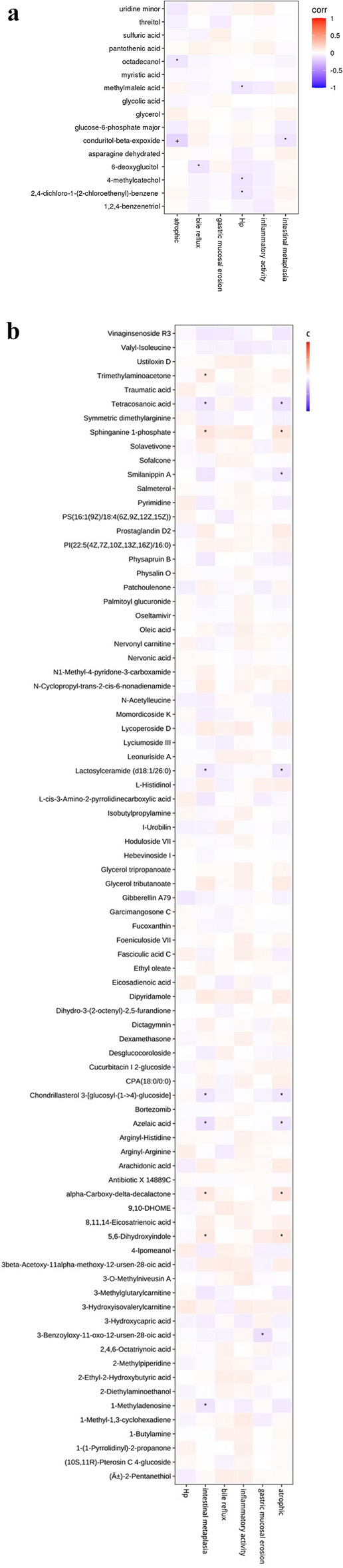
(**a**) Correlation figure between metabolic markers of tongue coating and gastroscopic indexes and pathological indexes in patients with chronic gastritis analyzed by GC-TOF-MS. Draw a thermodynamic diagram to show the correlation analysis results, which were represented by red (corr = 1), blue (corr = − 1) and white (corr = 0). Data with correlation P values less than 0.05 were marked with “*” in the figures. Data with correlation P values less than 0.01 were marked with “+” in the figures. The abscissa was the differential metabolic marker and the ordinate was the gastroscopic characteristic indicator. (**b**) Correlation figure between metabolic markers of tongue coating and gastroscopic indexes and pathological indexes in patients with chronic gastritis analyzed by UHPLC-QE-MS. Draw a thermodynamic diagram to show the correlation analysis results, which were represented by red (corr = 1), blue (corr = − 1) and white (corr = 0). Data with correlation P values less than 0.05 were marked with “*” in the figures. The abscissa was the differential metabolic marker, and the ordinate was the gastroscopic characteristic indicator.

As depicted in Fig. 4a, conduritol-beta-expoxide and octadecanol exhibited negative correlations with atrophy, while conduritol-beta-expoxide demonstrated a negative correlation with intestinal metaplasia. Moreover, 6-deoxyglucitol displayed a negative correlation with bile reflux, and 2,4-dichloro-1-(2-chloroethenyl)-benzene, 4-methylcatechol, and methylmaleic acid were negatively correlated with Hp infection. (Data with correlation P values less than 0.05 were marked with “*” in the figures. Data with correlation P values less than 0.01 were marked with “+” in the figures.)

Figure 4b reveals that, 5,6-Dihydroxyindole, alpha-Carboxy-delta-decalactone, Sphinganine1-phosphate, and Trimethylaminoacetone were positively correlated with intestinal metaplasia. Conversely, 1-Methyladenosine, Azelaic acid, Chondrillasterol 3-[glucosyl-(1->4)-glucoside], Lactosylceramide(d18:1/26:0), and Tetracosanoic acid were negatively correlated with intestinal metaplasia. Furthermore, 3-benzoyloxy-11-oxo-12-ursen-28-oic acid was negatively correlated with gastric mucosal erosion. 5,6-Dihydroxyindole, alpha-Carboxy-delta-decalactone, and Sphinganine1-phosphate were positively correlated with atrophy. Conversely, Azelaic acid, Chondrillasterol 3-[glucosyl-(1->4)-glucoside], Lactosylceramide(d18:1/26:0), Smilanippin A, and Tetracosanoic acid displayed negative correlations with atrophy. These correlations elucidate the potential associations between specific metabolites and various pathological features observed in CG patients. (Data with correlation P values less than 0.05 were marked with “*” in the figures.)

### Change trends of differential metabolite in different pathological stages

**Figure 5:**
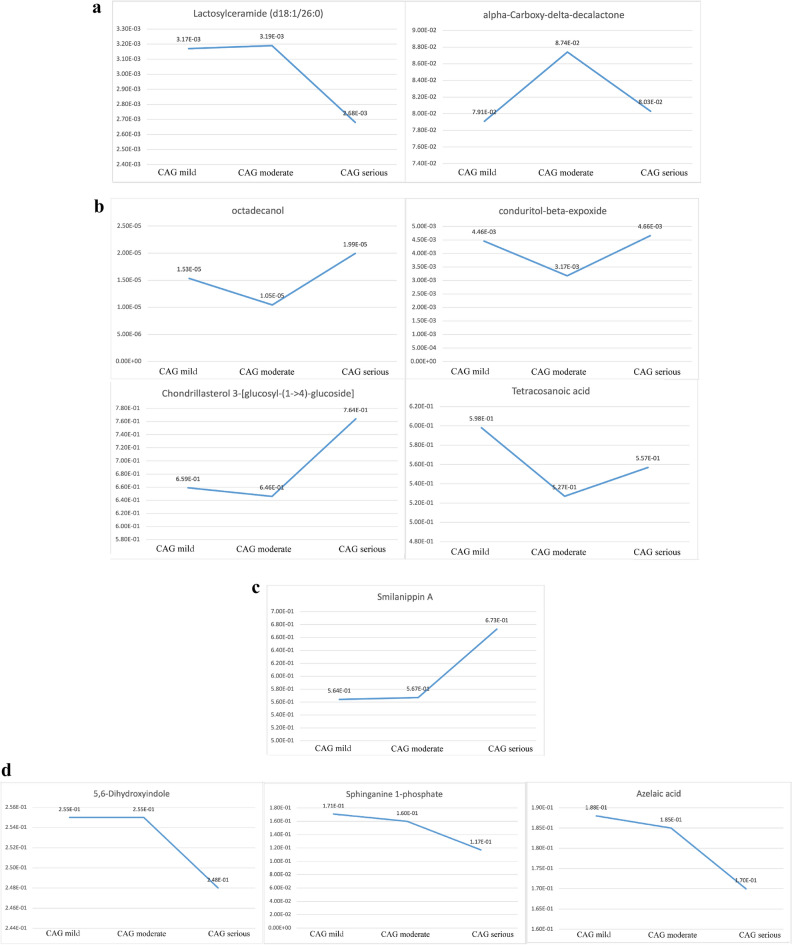
(**a**) Compounds with increasing and decreasing then content in atrophic gastritis of different degrees. The changing trend of Lactosylceramide and alpha-Carboxy-delta-decalactone (d18:1/26:0) content of atrophic patients was first increased and then decreased. (**b**) Compounds with decreasing and then increasing content in atrophic gastritis of different degrees. The changing trend of Octadecanol, Conduritol-beta-expoxide, Chondrillasterol 3-[glucosyl-(1->4)-glucoside], and Tetracosanoic acid contents of atrophic patients was first decreased and then increased. (**c**) Compounds with an increasing trend in content in atrophic gastritis of different degrees. The content of Smilanippin A in the tongue coating of atrophic patients showed an upward trend. (**d**) Compounds with a decreasing trend in content in atrophic gastritis of different degrees. The contents of 5,6-Dihydroxyindole, Sphinganine 1-phosphate, and Azelaic acid of atrophic patients showed a downward trend.

**Figure 6:**
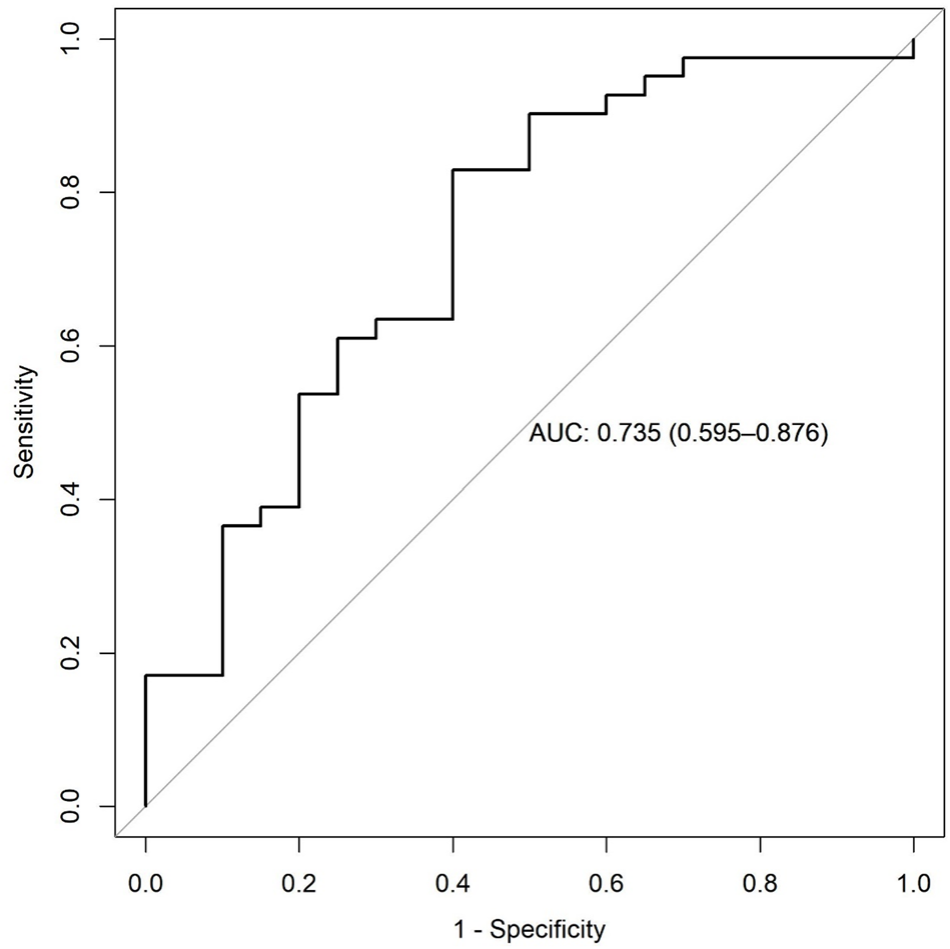
Diagnostic model of tongue coating metabolites in patients with with chronic superficial gastritis and chronic atrophic gastritis. The accuracy of the diagnostic model is 73.50%, the specificity is 60.00%, and the sensitivity is 82.93%.

According to Table 2, the contents of alpha-Carboxy-delta-decalactone, Azelaic acid, Chondrillasterol 3-[glucosyl-(1->4)-glucoside], Lactosylceramide (d18:1/26:0), and Octadecanol in the three stages of atrophic were lower than those in the healthy controls. The contents of 5,6-Dihydroxyindole, Conduritol-beta-expoxide, Smilanippin A, Sphinganine 1-phosphate, and Tetracosanoic acid in the three stages of atrophic were higher than those in the healthy controls. The content of conduritol-beta-expoxide decreased significantly in mild atrophic compared with moderate atrophic (*P* < 0.05). The changing trend of Lactosylceramide and alpha-Carboxy-delta-decalactone (d18:1/26:0) content of atrophic patients was first increased and then decreased (Fig. 5a). The changing trend of Octadecanol, Conduritol-beta-expoxide, Chondrillasterol 3-[glucosyl-(1->4)-glucoside], and Tetracosanoic acid contents of atrophic patients was first decreased and then increased (Fig. 5b). The content of Smilanippin A in the tongue coating of atrophic patients showed an upward trend (Fig. 5c). The contents of 5,6-Dihydroxyindole, Sphinganine 1-phosphate, and Azelaic acid of atrophic patients showed a downward trend (Fig. 5d). We further modelled random forests based our randomization combinations on metabolites with significant correlations. It was used to distinguish patients with chronic superficial gastritis 195 cases (no intestinal metaplasia) and those with atrophic gastritis 106 cases (some are accompanied by intestinal metaplasia). As shown in Fig. 6, the accuracy of the model was 75.3%, sensitivity was 82.9%, and specificity was 60.0%.

**Table 2:** The standardized peak areas of metabolites in the tongue coating of chronic gastritis patients at different pathological stages.

Metabolites	Mild mean	Moderate mean	Severe mean
**Atrophic**			
Octadecanol	1.534E−05	1.045E−05	1.994E−05
Conduritol-beta-expoxide	4.46E−03	3.17E−03*	4.66E−03
Tetracosanoic acid	5.98E−01	5.27E−01	5.57E−01
Sphinganine 1-phosphate	1.71E−01	1.60E−01	1.17E−01
Smilanippin A	5.64E−01	5.67E−01	6.73E−01
Lactosylceramide (d18:1/26:0)	3.17E−03	3.19E−03	2.68E−03
Chondrillasterol 3-[glucosyl-(1->4)-glucoside]	6.59E−01	6.46E−01	7.64E−01
Azelaic acid	1.88E−01	1.85E−01	1.70E−01
alpha-Carboxy-delta-decalactone	7.91E−02	8.74E−02	8.03E−02
5,6-Dihydroxyindole	2.55E−01	2.55E−01	2.48E−01
**Intestinal metaplasia**			
Conduritol-beta-expoxide	4.62E−03	3.20E−03*	4.66E−03
Trimethylaminoacetone	1.24E−01	1.16E−01	1.12E−01
Tetracosanoic acid	6.08E−01	5.24E−01	5.57E−01
Sphinganine 1-phosphate	1.64E−01	1.58E−01	1.17E−01
Lactosylceramide (d18:1/26:0)	3.51E−03	3.34E−03	2.68E−03
Chondrillasterol 3-[glucosyl-(1->4)-glucoside]	6.75E−01	6.56E−01	7.64E−01
Azelaic acid	1.84E−01	1.85E−01	1.70E−01
alpha-Carboxy-delta-decalactone	8.14E−02	8.77E−02	8.03E−02
5,6-Dihydroxyindole	2.54E−01	2.56E−01	2.48E−01
1-Methyladenosine	3.60E−01	3.92E−01	4.52E−01
**Hp infection**			
Methylmaleic acid	1.17E−02	1.19E−02	1.22E−02
4-methylcatechol	1.03E−02	1.13E−02	1.22E−02
2,4-dichloro-1-(2-chloroethenyl)-benzene	1.17E−02	1.13E−02	1.22E−02

*There were significant differences between mild and moderate, *P* < 0.05.

**Figure 7:**
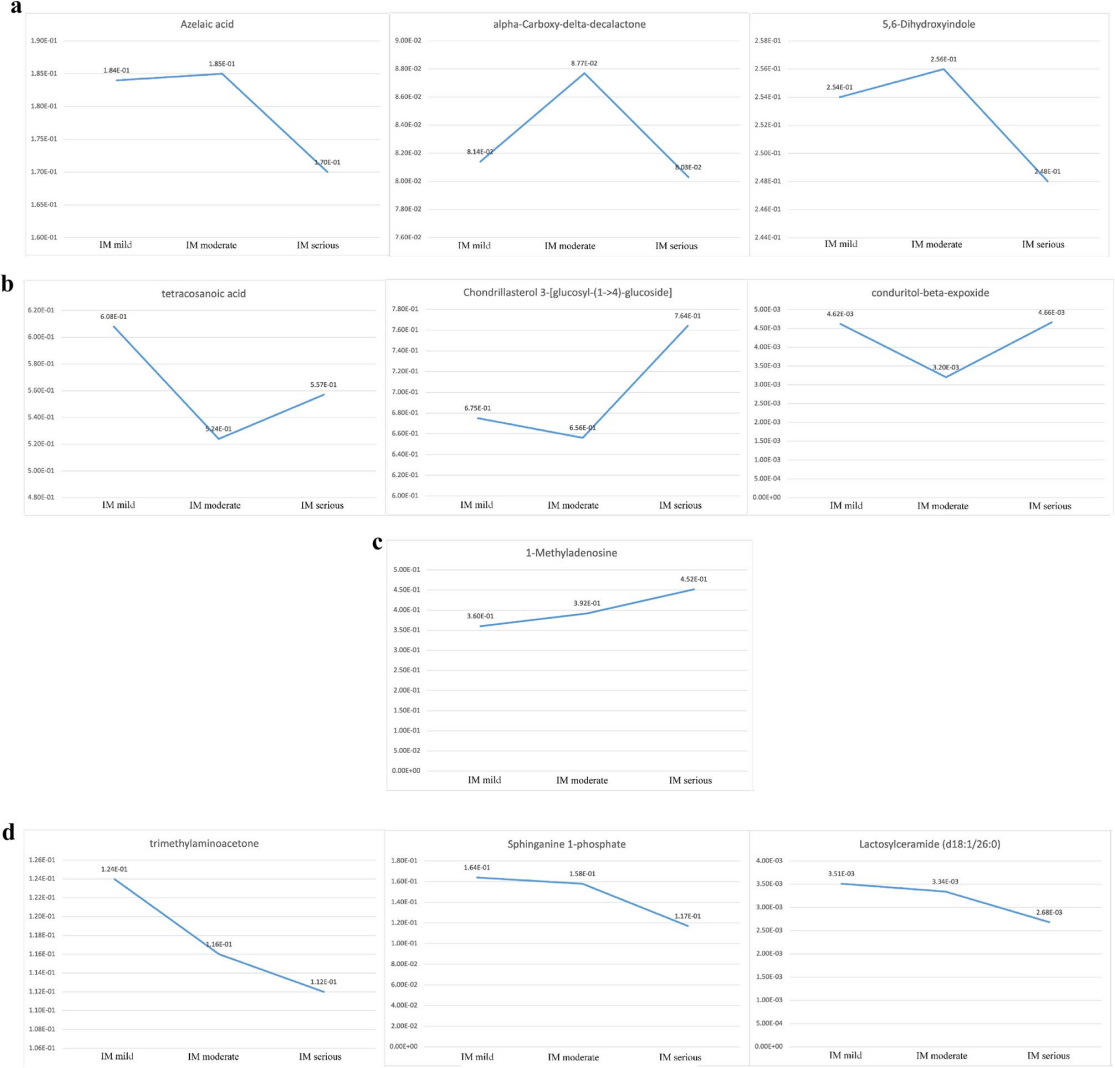
(**a**) Compounds with increasing and decreasing then content in intestinal metaplasia (IM) of different degrees. The changing trend of Azelaic acid, alpha-Carboxy-delta-decalactone, and 5,6-Dihydroxyindole contents of intestinal metaplasia patients was first increased and then decreased. (**b**) Compounds with decreasing and increasing then content in intestinal metaplasia (IM) of different degrees. The changing trend of Tetracosanoic acid, Chondrillasterol 3-[glucosyl-(1->4)-glucoside], and Conduritol-beta-expoxide contents of intestinal metaplasia patients was first decreased and then increased. (**c**) Compounds with an increasing trend in content in intestinal metaplasia (IM) of different degrees. The content of 1-Methyladenosine of intestinal metaplasia patients showed an upward trend. (**d**) Compounds with a decreasing trend in content in intestinal metaplasia (IM) of different degrees. The contents of Trimethylaminoacetone, Sphinganine 1-phosphate, and Lactosylceramide (d18:1/26:0) of intestinal metaplasia patients showed a downward trend.

In intestinal metaplasia patients, the contents of 1-Methyladenosine, alpha-Carboxy-delta-decalactone, Azelaic acid, Chondrillasterol 3-[glucosyl-(1->4)-glucoside], and Lactosylceramide (d18:1/26:0) in the three stages of intestinal metaplasia were lower than those in healthy controls. The contents of 5,6-Dihydroxyindole, Conduritol-beta-expoxid, Sphinganine 1-phosphate, Tetracosanoic acid, and Trimethylaminoacetone in the three stages of intestinal metaplasia were higher than those in healthy controls. The content of Conduritol-beta-expoxide decreased significantly in mild intestinal metaplasia compared with moderate intestinal metaplasia (*P* < 0.05). The changing trend of Azelaic acid, alpha-Carboxy-delta-decalactone, and 5,6-Dihydroxyindole contents of intestinal metaplasia patients was first increased and then decreased (Fig. 7a). The changing trend of Tetracosanoic acid, Chondrillasterol 3-[glucosyl-(1->4)-glucoside], and Conduritol-beta-expoxide contents of intestinal metaplasia patients was first decreased and then increased (Fig. 7b). The content of 1-Methyladenosine of intestinal metaplasia patients showed an upward trend (Fig. 7c). The contents of Trimethylaminoacetone, Sphinganine 1-phosphate, and Lactosylceramide (d18:1/26:0) of intestinal metaplasia patients showed a downward trend (Fig. 7d).

**Figure 8:**
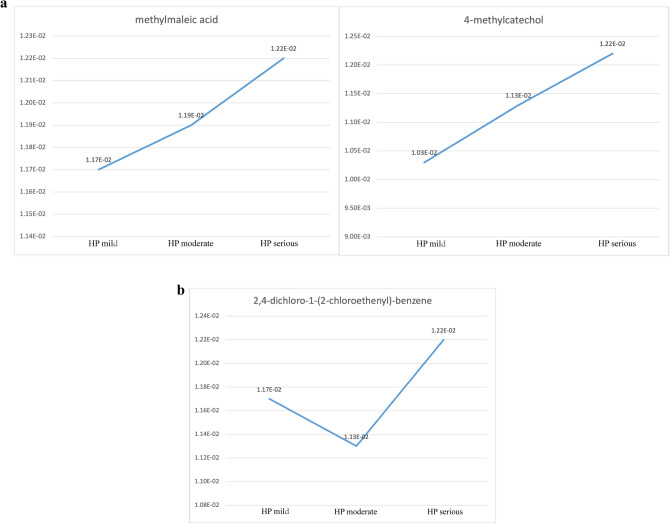
(**a**) Compounds with an increasing trend in content in HP of different degrees. The contents of Methylmaleic acid and4-methylcatechol of Hp infection patients showed an upward trend. (**b**) Compounds with decreasing and increasing then content in HP of different degrees. The changing trend of 2,4-dichloro-1-(2-chloroethenyl)-benzene content of Hp infection patients was first decreased and then increased.

In Hp infection patients, the contents of 2,4-dichloro-1-(2-chloroethenyl)-benzene, 4-methylcatechol, and Methylmaleic acid in the three stages of Hp infection were higher than those in the healthy controls. The contents of Methylmaleic acid and 4-methylcatechol of Hp infection patients showed an upward trend (Fig. 8a). The changing trend of 2,4-dichloro-1-(2-chloroethenyl)-benzene content of Hp infection patients was first decreased and then increased (Fig. 8b).

## Discussion

We detected the tongue coating metabolites of CG patients by metabolomics, compared with the controls, the number of lipids and lipid-like molecules of CG patients was the largest (there were 47 substances in total, of which 22 up regulated and 25 down regulated), accounting for 55.29% of the total metabolites. Researchers have found that Lipid oxidative stress can lead to a variety of chronic inflammatory diseases^14^.

Some researchers also have found that lipids and lipid-like molecules as the main different serum metabolites in the CG patients may promote the development of CG to gastric cancer. This study^15^ focuses on the diagnostic significance of lipid metabolites in serum. Linoleamide, which is mentioned in the paper as a metabolite upregulated in both chronic gastritis (CG) and gastric cancer (GC) patients, was also observed to change in the tongue coating of CG patients. Sphinganine 1-phosphate, identified as a candidate biomarker in the paper, was also found in our study to have increased levels in CG patients and is correlated with pathological indicators. However, our study involves a richer variety of lipid compounds with greater abundance variations. We speculate that the microbial metabolism in the oral cavity and digestive tract may affect the types and abundance of lipid compounds, leading to significant differences from those found in serum. Further research is needed to confirm this hypothesis.

Among the different metabolites of lipids and lipid-like molecules there was a substance called Sphinganine 1-phosphate, which was significantly elevated compared to healthy individuals. Sphinganine 1-phosphate was one of the markers of the diagnostic model of chronic gastritis. It has also been found to have significant correlation with atrophy and intestinal metaplasia which was positive. Studies have also shown that Sphinganine 1-phosphate may inhibition of apoptosis^16^, It can also significantly inhibit liver necrosis and cell apoptosis caused by liver ischemia–reperfusion^17^. In addition, we found that the level of Sphinganine 1-phosphate decreased gradually with the severity increases of gastric mucosal atrophic and intestinal metaplasia in CG patients. We speculated that the decrease of this substance may be related to the aggravation of the disease. Among the different metabolites screened by the diagnostic model of CG, except Sphinganine 1-phosphate, there was another substance belonging to the different metabolites of lipids and lipid-like molecules, which was named Nervonic acid. Some researchers found Nervonic acid in the mucosa of gastric cancer patients^18^. Nervonic acid exists not only in the upper digestive tract but also in the lower digestive tract. Moreover, as one kind of lipid, Nervonic acid can increase fatty acid oxidation in the liver and reduce the level of circulating triglycerides leading to obesity^19^.

As shown in Fig. 5c the content of Smilanippin A of severe atrophic patients was higher than that of mild and moderate atrophic patients. The content of Lactosylceramide (d18:1/26:0) of severe atrophic patients was lower than that of mild and moderate atrophic patients. In addition, according to Fig. 7d, the content of Lactosylceramide (d18:1/26:0) gradually decreased with the aggravation of intestinal metaplasia. Lactosylceramide can promote the growth of colon cancer cells^20^, and such substance also exists in the serum of children with inflammatory bowel disease^21^. This substance will aggravate digestive system diseases. Moreover, oxidative stress environment can promote inflammation^22^. Glycosphingolipids readily lead to the occurrence of free radical induced oxidation, this oxidation may be related to the increase of lactosylceramide content^23^.

In Figs. 5b and 7b, the content of Chondrillasterol 3-[glucosyl-(1->4)-glucoside] of severe atrophic and intestinal metaplasia patients was higher than that of patients with mild and moderate atrophic and intestinal metaplasia. Moreover, the content of this substance in the three stages of atrophic and intestinal metaplasia was lower than that in the tongue coating of healthy controls. The content of Azelaic acid gradually decreases with the aggravation of atrophic, and its content in the tongue coating of subjects with different degrees of atrophic was lower than healthy people. Moreover, the content of Azelaic acid in severe intestinal metaplasia subjects was lower than that in mild and moderate patients, and the content in the three stages of intestinal metaplasia was lower than that in the tongue coating of healthy controls. Azelaic acid is a marker of lipid peroxidation^24^. Researchers found that the content of Azelaic acid in the plasma of gastric cancer patients was significantly increased^25^. However, we found that the content of Azelaic acid in the tongue coating of gastric precancerous lesion patients was lower than healthy people. This phenomenon deserves our in-depth study in the future.

Organic acids and derivatives were the second largest metabolites. There were 13 metabolites (7 up regulated and 6 down regulated), among which pantothenic acid was up regulated. Some researchers have found that this substance in gastric cancer was a potential biomarker^26^. The different metabolite 3-Hydroxycapric acid screened by the diagnostic model of CG also belongs to Organic acids and derivatives.

In other metabolic species, l-histidinol expression was up regulated. Some studies have shown that histidinol can inhibit gastric acid secretion and alleviate stomach pain. In addition, it can also inhibit the growth and metastasis of lung cancer cells^27,28^. Dictagymnin expression was up regulated, and this substance has anti-inflammatory and tumor growth inhibitory effects^29^. Patients with chronic gastritis were probably undergoing self-repair by the body during their illness. From the correlation between chronic gastritis and pathological indexes, we found that the differential metabolites belonging to Organoheterocyclic compounds were 5,6-Dihydroxyindole and alpha-Carboxy-delta-decalactone. In Figs. 5d and 7a, the content of 5,6-Dihydroxyindole of severe atrophic and intestinal metaplasia patients was lower than that of patients with mild and moderate atrophic. The content of alpha-Carboxy-delta-decalactone of moderate atrophic and intestinal metaplasia patients was higher than that of patients with mild and severe atrophic.

Among the different metabolites screened by the diagnostic model of chronic gastritis, 4-Ipomeanol belongs to Organic oxygen compounds, and 1-Methyladenosine belongs to Nucleosides, nucleotides, and analogues. 1-Methylladenosine was also found in the results of correlation of pathological indexes. In the trend figures of chronic gastritis with intestinal transformation, the content of 1-Methylladenosine increased with the increase of intestinal degree.

In addition, as shown in Fig. 8a. The contents of methylmaleic acid and 4-methylcatechol gradually increased with the aggravation of Hp infection. The content of 2,4-dichloro-1-(2-chloroethenyl)-benzene of moderate intestinal metaplasia patients was lower than mild and severe patients. Experiments on rats showed that 4-methylcatechol and Hp infection may cause gastric cancer^30,31^. We suggest that 4-methylcatechol might be relate to the transformation from CG with Hp infection to gastric cancer. In Table 2, we also found that the content of Conduritol-beta-expoxide decreases significantly with the severity increases of atrophic and intestinal metaplasia. However, we have not found the content change of this substance in digestive system diseases.

With further investigation, these metabolites present in the tongue coatings of CG patients hold promise as potential non-invasive diagnostic markers, augmenting gastroscope-based diagnosis and monitoring of patients with chronic gastritis. Continued research in this area may offer valuable insights into improving diagnostic methods and enhancing patient care for CG.

## Supplementary Information

Supplementary Information.

## Data Availability

In the study, experimental data other than personal information of the subjects can be obtained by contacting the corresponding author.
