# Supplementary material for: The metabolites mainly composed of lipids in tongue coating are non-invasive potential biomarkers for chronic gastritis
Source: Sci Rep. 2024 Jul 30;14:17574. doi: 10.1038/s41598-024-68261-6 (PMC11289369; doi:10.1038/s41598-024-68261-6)
Supplement: Supplementary file 1 — Supplementary Information. [file 41598_2024_68261_MOESM1_ESM.docx]

**1. Inclusion and exclusion criteria**

The inclusion criteria were as follows (See Figure S1 for the specific process):

1) The patients met the diagnostic criteria of CG and the healthy controls had no evidence of systemic organic lesions.

2) The participants’ age range was from 20 to 70 years.

The exclusion criteria were as follows:

1) The patients had gastroesophageal reflux disease, gastric ulcer, gastric hemorrhage, gastric cancer, duodenal ulcer, or other intestinal diseases through endoscopic examination.

2) The patients had diseases of the motor system, nervous system, endocrine system, circulatory system, respiratory system, urinary system and reproductive system.

3) The participants had mental illness.

4) Female subjects were during their pregnancy and lactation period.

5) The participants had lesions of the tongue, mouth, nose or pharynx within one month prior to the collection of samples.

6) The participants had received probiotics or antibiotics within one month prior to sample collection.

7) The participants used tobacco or consumed alcohol.

8) The participants had a body mass index (BMI) over 28.

**2. Tongue coating samples collection**

Samples of tongue coating were collected before participants had breakfast. Before collection, the participants rinsed the mouth with a stroke-physiological saline solution 3 times in order to remove the residues in the mouth. The collector used a sterile specimen collection swab (CY-98000, iClean, Huachenyang Technology Co., Ltd, CN) to scrape the thick part of the tongue coating of participants 5 times, then put the head of the tongue coating sample swabs into a sterile centrifuge tube. All tongue coating samples were collected by the same researcher. The tongue coating samples were stored for use at -80°C.

**3. GC-TOF-MS Metabolomics Processing**

1) The swab head of each tongue sample was individually transferred to a 5 ml sterile Eppendorf (EP) tube and weighed, then pre-cooled extraction mixture (V methanol: V chloroform = 3:1) 1500 μL was added, and the internal standard solution (alditol, 0.5 mg/ml stock solution) 15 μL. The samples were sonicated in iced water for 30 minutes. The swab head was then removed. After centrifugation at 4°C and 10,000 rpm for 15 minutes, 500 μL of supernatant was transferred to a fresh tube.2) We took 100 μL from each sample and combined it together to prepare a quality control (QC) sample. After evaporation in a vacuum concentrator, add 40 μL methoxyaminated hydrochloride (20 mg/ml pyridine), incubated at 80°C for 30 min, and then incubated with 60 ml at 70°C μL bis-(trimethylsilyl)-trifluoroacetamide regent (containing 1% trimethylchlorosilane, V/V) was derived for 1.5h. After gradually cooled the sample to room temperature, 5 μL fatty acid methyl ester (in chloroform) was added to QC samples.

3) GC-TOF-MS detection was performed using an Agilent 7890 gas chromatograph and TOF mass spectrometer. The system employed a db-5ms capillary column. Inject 1 μL aliquot in splitless mode. With Helium as the carrier gas, the purge flow at the front inlet was 3 mL/min, and the gas flow rate through the chromatographic column was 1 mL/min. The initial temperature was maintained at 50°C for 1 min, and then raised to 310°C at a rate of 20°C/min, then maintained at 310°C for 6 min. The implantation, the transfer line, and ion source temperatures were 280, 280, and 250°C. The energy was -70eV in electron collision mode. The mass spectrum data were obtained in full-scan mode with the m/z range of 50-500 at a speed of 12.5 spectrum/s and solvent delay of 6.30 min.

**4. UHPLC-QE-MS Metabolomics Processing**

1) The swab head of each tongue sample was individually transferred to a 5 ml sterile Eppendorf (EP) tube.Add 1500 μL of extract containing the isotopically-labeled internal standard mixture (Vacetonitrile:Vmethanol: Vwater = 2:2:1). After being vortexed for 30s, the solution was sonicated on ice for 30 min.

2) - 40°C for 1 h and centrifuged at 12000 rpm for 15 minutes at 4°C. The resulting supernatant was transferred to a fresh glass vial for analysis. The supernatants of all the samples were mixed well to make QC samples.

3) LC-MS detection was performed on a UHPLC system (Vanquish, Thermo Fisher Scientific) with a UPLC BEH Amide column (2.1 mm×100 mm, 1.7 μm) coupled to Q Exactive HFX mass spectrometer (Orbitrap MS, Thermo). The mobile phases were 25 mmol/L ammonium acetate and 25 mmol/L ammonia hydroxide in water (pH = 9.75) (A) and acetonitrile (B). The elution gradient as follows: 0-0.5 min, 95% B; 0.5-7.0 min, 95%-65% B; 7.0-8.0 min, 65%-40% B; 8.0-9.0 min, 40% B; 9.0-9.1 min, 40%-95% B; 9.1-12.0 min, 95% B. The column temperature was 25°C. The auto-injection temperature was 4°C, and the injection volume was 3 μL.

4) The QE HFX mass spectrometer acquired MS/MS spectra in the information-dependent acquisition mode under the control of the acquisition software (Xcalibur, Thermo). In this mode, the acquisition software continuously assessed the full scan mass spectrum. The ESI source conditions were set as following: sheath gas flow 50 Arb, auxiliary gas flow 10 Arb, capillary temperature 320°C, full MS resolution as 60000, MS/MS resolution as 7500, collision energy 10/30/60 in nice mode, spray Voltage 3.5 kV (positive) or -3.2 kV (negative), respectively.

5) The raw data were converted to the mzXML format using ProteoWizard and peak detection, extraction, alignment, and integration were performed using an in-house XCMS based program developed in R.

6) Metabolite annotation was adopted from the company’s own MS2 database (BiotreeDB V2.1).

**Table S2. Summary of demographics and clinical information of the participants**

| Demographics and clinical information | Chronic gastritis patient group | Healthy control group |
| --- | --- | --- |
| Sample number | 350 | 50 |
| Ratio of male to female | 1:0.74 | 1:0.92 |
| Average age (year) | 45.64±13.62 | 35.42±9.82 |
| Number (percentage) of samples diagnosed this time | 3 (0.86%) | N/A |
| Number (percentage) of samples diagnosed for less than 10 years | 259 (74.00%) | N/A |
| Number (percentage) of samples diagnosed for 10-20 years | 71 (20.29%) | N/A |
| Number (percentage) of samples diagnosed for 20-30 years | 15 (4.29%) | N/A |
| Number (percentage) of samples diagnosed for 30-40 years | 4 (1.14%) | N/A |
| Number (percentage) of samples diagnosed over40 years | 1 (0.29%) | N/A |
| Number (percentage) of samples untreated | 84 (24.00%) | N/A |
| Number (percentage) of samples only taking western medicine | 86 (24.57%) | N/A |
| Number (percentage) of samples only taking traditional Chinese medicine | 92 (26.29%) | N/A |
| Number (percentage) of samples taking western medicine and traditional Chinese medicine | 88 (25.14%) | N/A |

**Table S2.** **Distribution of severity in patients with chronic gastritis in different indexes of gastroscopic observation and gastric mucosal pathology**

| **indexes of gastroscopic observation and gastric mucosal pathology** | **Mild** | **Moderate** | **Severe** | **Proportion of total samples,** **%** |
| --- | --- | --- | --- | --- |
| inflammatory activity | 48 | 45 | 3 | 96 (27.43%) |
| atrophic | 101 | 22 | 6 | 129 (36.86%) |
| intestinal metaplasia | 87 | 21 | 6 | 114 (32.57%) |
| Hp infection | 10 | 20 | 19 | 49 (14.00%) |
| bile reflux | N/A | N/A | N/A | 60 (17.14%) |
| gastric mucosal erosion | N/A | N/A | N/A | 171 (48.86%) |

**Table S3. Reagents used in the experiments**

| Reagents | Manufacturer |
| --- | --- |
| ***a. Reagents used in GC-TOF-MS experiment*** | |
| Methanol | CNW Technologies, Germany |
| Chloroform | Adamas, Switzerland |
| Pyridine | Adamas, Switzerland |
| Methoxy amination hydrochloride | Tokyo Chemical Industry, Japan |
| Adonitol | Sigma-Aldrich, USA |
| Bis-(trimethylsilyl)-trifluoroacetamide (with 1% trimethylchlorosilane, v/v) | Regis Technologies, USA |
| Fatty acid methyl esters | Dr. Ehrenstorfer, Germany |
| ***b. Reagents used in UHPLC-QE-MS experiment*** | |
| Methanol | CNW Technologies, Germany |
| Acetonitrile | CNW Technologies, Germany |
| Ammonium acetate | CNW Technologies, Germany |
| Ammonium hydroxide | CNW Technologies, Germany |

**
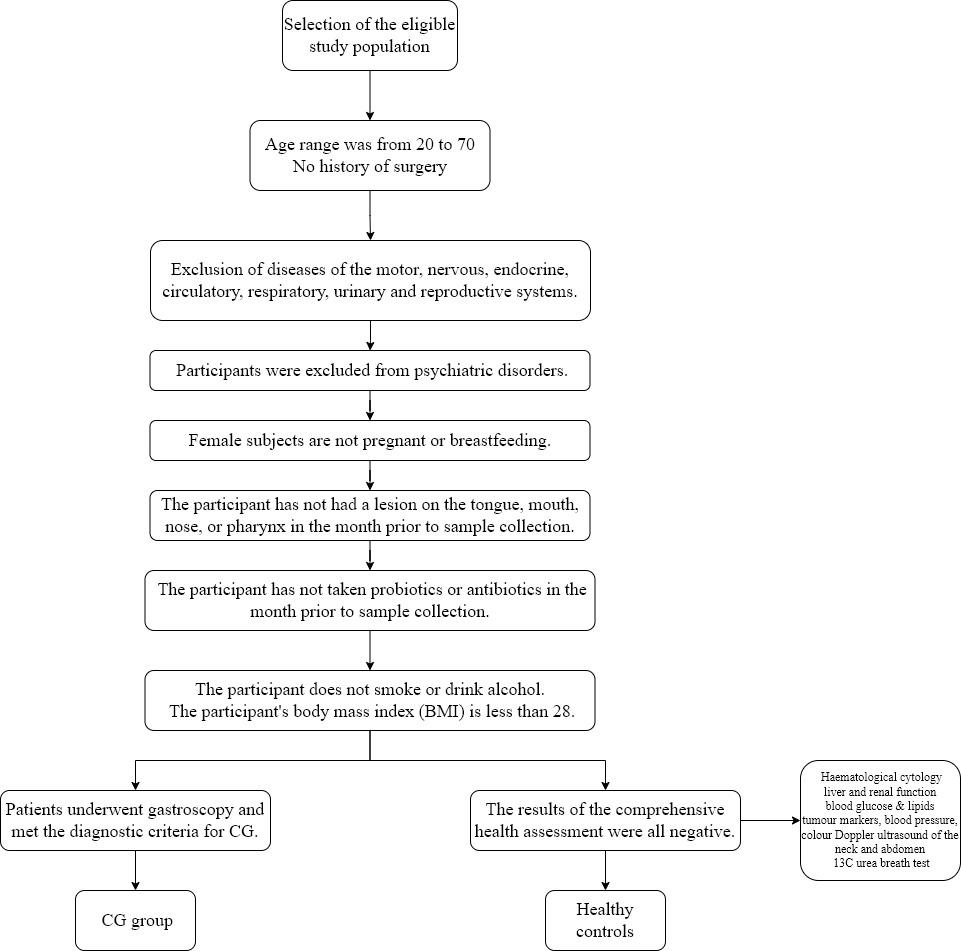
**

**Figure S1.** **Flowchart for selection of eligible study populations**

The figure shows the screening process for participants in the healthy and CG groups.


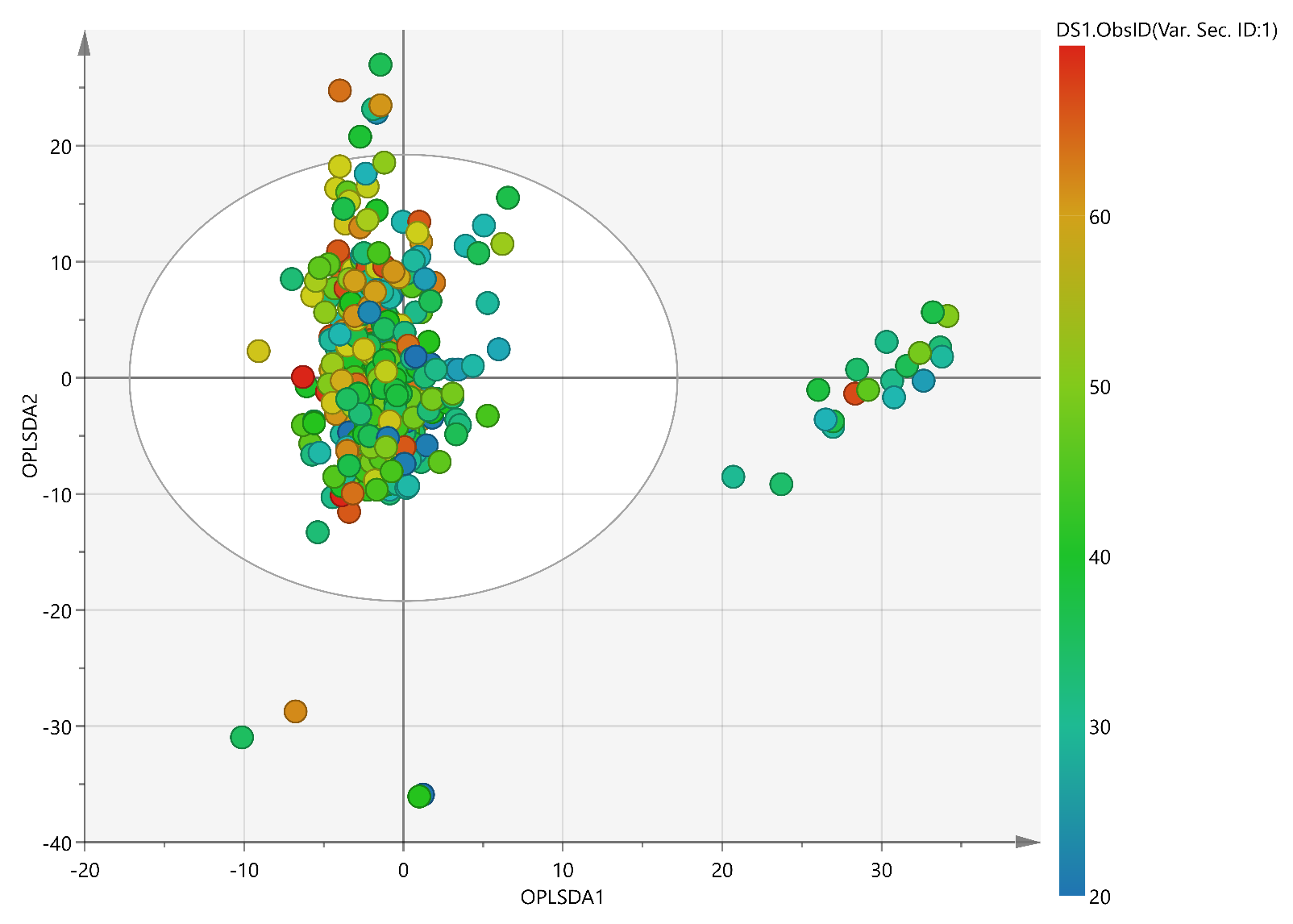


**Figure S2-a**


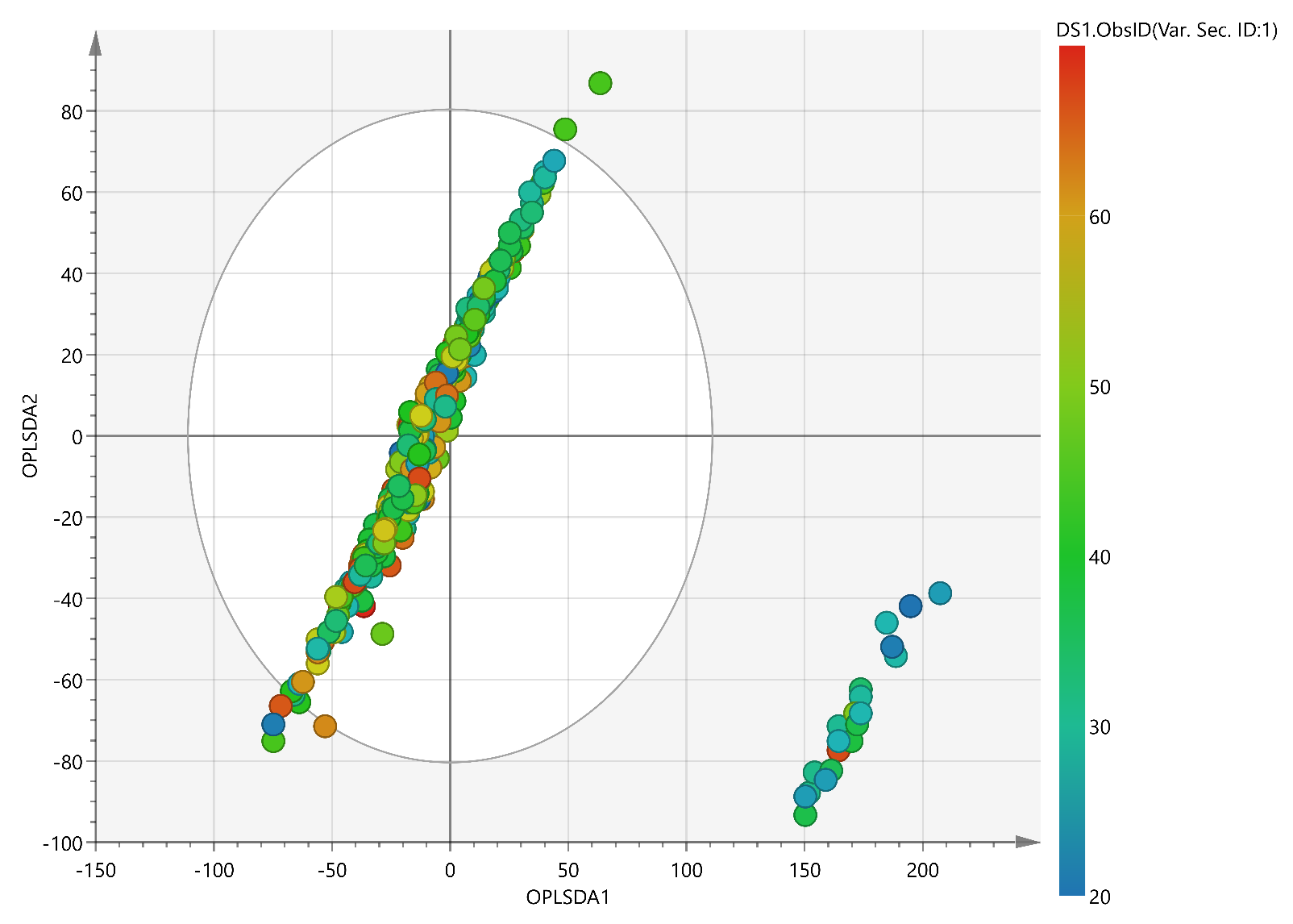


**Figure S2-b**


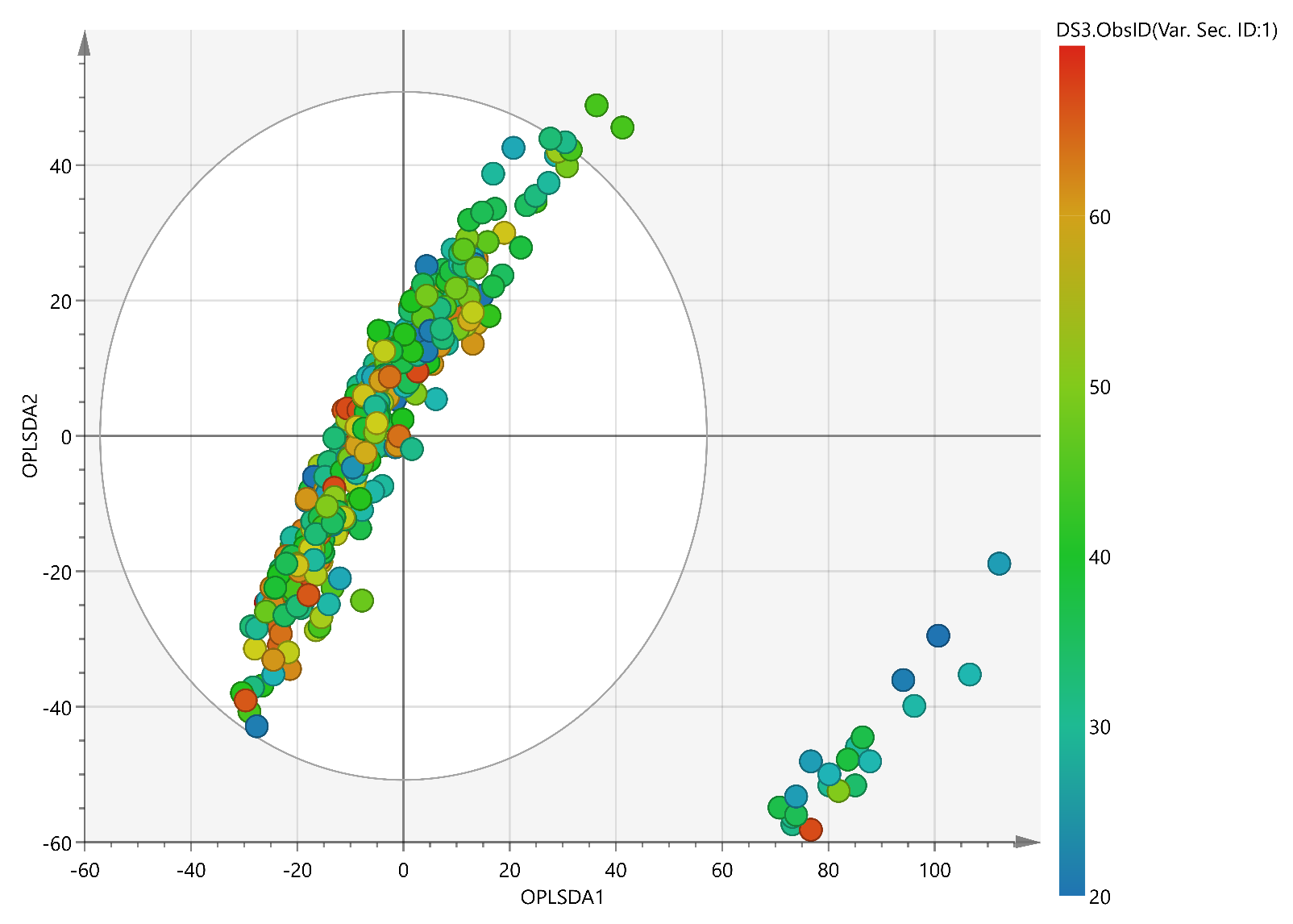


**Figure S2-c**

**Figure S2. OPLS-DA reflecting the impact of age factors on different metabolites of chronic gastritis group and healthy control group**

a: GC-TOF-MS, b: UHPLC-QE-MS negative ion modes, c: UHPLC-QE-MS positive ion modes.

As the patients’ age changed, the metabolites of the two groups of samples were not significantly distinguished.
